# Exploratory study on the mechanism of necrotic effect of nourishing cells in the context of genital tract infection in premature rupture of membranes

**DOI:** 10.1097/MD.0000000000036148

**Published:** 2023-12-15

**Authors:** Yunying Qian, Guiying Qian, Haiyan Ni, Danying Zhu, Weiqun Gu, Ximei Cai

**Affiliations:** a Department of Obstetrics and Gynecology, Changshu Hospital Affiliated to Nanjing University of Chinese Medicine, Nanjing, Jiangsu, China; b Department of Pharmacy, Changshu Hospital Affiliated to Nanjing University of Chinese Medicine, Nanjing, Jiangsu, China.

**Keywords:** genital tract infection, necrotic effect, nourishing cells, premature rupture of membranes

## Abstract

To explore the mechanism of necrotic effect of nourishing cells in the context of genital tract infection in premature rupture of membranes (PROM). One hundred eight patients with PROM treated at our hospital from June 2020 to June 2022 were selected as the PROM group. Simultaneously, 108 cases of normal full-term pregnant women were chosen as the control group. Western blot analysis was performed to measure the relative expression levels of cysteinyl aspartate specific proteinase-1 (Caspase-1), cysteinyl aspartate specific proteinase-3 (Caspase-3), nucleotide-binding oligomerization domain-like receptor family pyrin domain containing 3 (NLRP3), and interleukin (IL)-1β proteins, which are associated with necrosis of placental nourishing cells, in the placenta of both groups. TUNEL staining was used to detect the number of apoptotic placental nourishing cells. The differences in necrotic factors of placental nourishing cells were analyzed between full-term and preterm cases in the PROM group, as well as among patients with different genital tract infections. The apoptotic count of placental nourishing cells in the PROM group was 58.46 ± 11.26 cells/field, which was markedly higher than that of the control group (*P* < .05). The relative expression levels of the necrotic factors Caspase-1, Caspase-3, NLRP3, and IL-1β proteins in placental nourishing cells of the PROM group were 1.32 ± 0.26, 1.19 ± 0.30, 1.29 ± 0.28, and 1.23 ± 0.24, respectively. These values were significantly higher than those of the control group (*P* < .05). The relative expression levels of the necrotic factors Caspase-1, Caspase-3, NLRP3, and IL-1β proteins in placental nourishing cells were compared between full-term and preterm patients in the PROM group (*P* > .05). The relative expression levels of the necrotic factors Caspase-1, Caspase-3, NLRP3, and IL-1β proteins in placental nourishing cells were higher in patients with multiple genital tract infections compared to those with single infections or no infections in the PROM group (*P* < .05). PROM is associated with a significant upregulation of placental nourishing cell apoptosis and necrotic factors, including Caspase-1, Caspase-3, NLRP3, and IL-1β proteins. This upregulation is correlated with the presence of genital tract infections.

## 1. Introduction

Premature rupture of membranes (PROM) is a common obstetric complication characterized by the rupture of fetal membranes before the onset of labor.^[1]^ Research has consistently demonstrated that PROM is associated with a significantly higher risk of perinatal mortality compared to cases where the fetal membranes remain intact, underscoring the importance of its clinical management.^[2,3]^ Based on the gestational age at which it occurs, PROM can be categorized as either preterm PROM or term PROM. Investigating the underlying causes of PROM is of paramount significance for enhancing maternal and neonatal outcomes.^[4,5]^ Currently, the precise etiology of PROM remains incompletely understood. Existing research suggests that multiple factors, including infection, abnormal intrauterine pressure, nutritional factors, psychosocial influences, genetics, and apoptosis, are implicated in the pathogenesis of PROM.^[6–8]^ Notably, recent studies have demonstrated the involvement of fractalkinin, a potent proinflammatory factor, in the development of PROM.^[9]^ Furthermore, investigations have revealed increased activation of inflammasomes, including NLRP1, nucleotide-binding oligomerization domain-like receptor family pyrin domain containing 3 (NLRP3), AIM2, and NLRC4, in cases of PROM. Of particular note, the ADAMTS4 level is further elevated in the PROM group and exhibits a significant correlation with inflammasome expression. The heightened expression of NLRC4 in fetal membranes may contribute to the recruitment of more cysteinyl aspartate specific proteinase-1 (caspase-1), thereby amplifying the inflammatory response in PROM patients. Given that the pathogenesis of early membrane PROM is closely associated with inflammatory responses, inflammasome-mediated caspase-1 activation plays a pivotal role in the secretion of downstream pro-inflammatory cytokines such as interleukin-1β (IL-1β) and IL-18.^[10,11]^ Consequently, there is a significant increase in the levels of NLRPs, IL-1 family members, and Caspases in fetal placental tissue of the premature rupture group. Some studies have postulated that genital tract infection is a key triggering factor for PROM. Notably, necroptosis, a form of inflammatory cell death with similarities to apoptosis, has garnered attention in this context. Substantial research has highlighted that trophoblast necroptosis can lead to placental abnormalities.^[12,13]^ Nevertheless, limited research has explored the mechanistic role of trophoblast necroptosis in the context of PROM and genital tract infection. To bridge this knowledge gap, this study aims to gather data from pregnant women affected by PROM and genital tract infection. Specifically, the expression of necroptosis-related molecules in their placental tissue will be examined, providing valuable insights for elucidating the pathogenesis of cell necroptosis in this context.

## 2. Data and methods

### 2.1. General data

The study was approved by the Ethics Committee of Changshu Hospital Affiliated to Nanjing University of Chinese Medicine. The study selected 108 cases of patients with PROM who were treated at our hospital from June 2020 to June 2022 as the PROM group. The age of the patients ranged from 20 to 36 years, with a mean age of 29.41 ± 2.66 years. Additionally, 108 cases of normal full-term pregnant women were selected as the control group, with an age range of 21 to 36 years and a mean age of 28.22 ± 3.03 years. The ages of the PROM group and the control group were compared (*P* > .05). Inclusion criteria: (1) The diagnosis of PROM meets the diagnostic criteria in “Obstetrics and Gynecology.” (2) 20 to 36 years old. (3) Singleton pregnancy, head position. (4) 28 to 41 gestational weeks. (5) Informed consent of patients and their families. Exclusion criteria: (1) Combined with malignant tumors, liver and kidney dysfunction, and other serious diseases. (2) Have a history of drug abuse. (3) No complications such as gestational diabetes mellitus and gestational hypertension. (4) There was a history of use of antibiotics and glucocorticoids in the past 1 month. Among the 106 patients in the infection group, 10 (9.4%) were Ureaplasma urealyticum, 37 (44.3%) were group B streptococcus, 7 (6.6%) were anaerobe, 5 (4.7%) were chlamydia, 27 (25.5%) were mixed infected, and 20 (18.8%) were no infected.

### 2.2. Western blot detection

The protein levels of Caspase-1, cysteinyl aspartate specific proteinase-3 (Caspase-3), NLRP3, and IL-1β were measured. After delivery, 1.0 to 2.0 grams of placental tissue were collected from the postpartum women. Total protein was extracted from the placental tissue, and the protein concentration of the aforementioned markers were determined using the bicinchoninic acid assay method. Following the instructions, denaturation, membrane preparation, and sample loading were carried out (30 μg of protein per well). Sodium dodecyl sulfate polyacrylamide gel electrophoresis gel electrophoresis was performed with the stacking gel run at 80 V for 40 minutes and the separating gel run at 110 V for 60 minutes. After electrophoresis, the proteins were transferred onto a Poly(vinylidene fluoride) membrane (Merck) using a constant current of 300 mA. The transferred membrane was incubated with a 5% skim milk powder blocking solution for 1 hour, followed by the addition of primary antibodies against Caspase-1, Caspase-3, NLRP3, and IL-1 β (Abcam). The membrane was incubated overnight at 4°C, followed by washing. Then, secondary antibodies were added to the membrane and incubated at room temperature for 1 hour. After washing the membrane, ECL reagent (Tanon) was used for chemiluminescent detection. The images were obtained for subsequent statistical analysis.

### 2.3. Apoptosis of placental trophoblast cells by TUNEL detection

The placental tissue was collected and processed for routine preparation of paraffin sections. Deparaffinization was performed. Dojindo apoptosis assay kit has been applied. Proteinase K was diluted 1:200 in 0.01 mmol/L TBS and incubated at 37°C for 30 minutes for digestion. The sections were washed 3 times with TBS. To prepare the TUNEL detection solution, TdT, DIG-dUTP, and labeling buffer were mixed in a ratio of 1:1:18. For each tissue section, 20 μL of the detection solution was applied. The sections were incubated at 37°C for 2 hours, followed by 3 washes with TBS. Then, the sections were blocked for half an hour. Subsequently, the sections were incubated and washed using biotinylated anti-digoxigenin antibody and SABC solution under the same conditions. Diamidino-2-phenylindole staining was performed, and the slides were coverslipped. The sections were observed under a fluorescence microscope at 400× magnification. Nine different fields of view were randomly selected on each slide. The number of positive cells was counted in each field, and the average value was calculated.

### 2.4. Statistical processing

SPSS22.0 software was used. The measurement data included age, IL-1β, Caspase-1, etc. The data were expressed as ($\bar{x}\pm s$), and the difference between the groups was analyzed by *t* test. *P* < .05 indicated that the difference was statistically significant.

## 3. Results

### 3.1. Comparison of trophoblast cell apoptosis in the placenta between the PROM group and the control group

**Figure 1.**
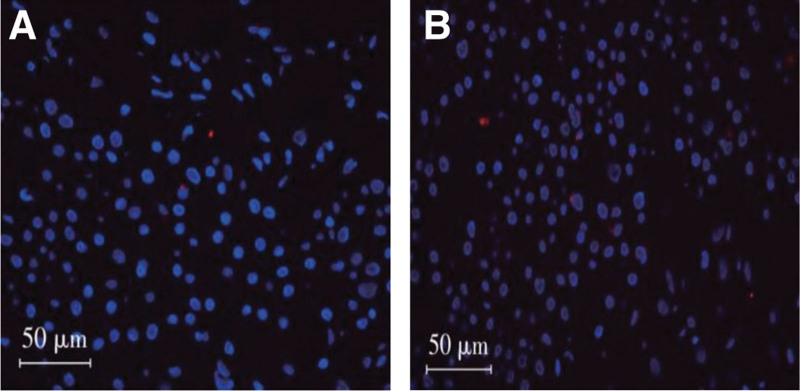
TUNEL of pyroptosis of placental trophoblast in 2 groups.

Indeed, the number of trophoblast cell apoptosis in the placenta was notably higher in the PROM group when compared to the control group, demonstrating a statistically significant difference (*P* < .05). For more comprehensive data, kindly consult Table 1. Figure 1 shows 2 groups of TUNEL with placental trophoblast pyrosis.

**Table 1:** Comparison of trophoblast cell apoptosis in the placenta between the PROM group and the control group.

Group	Number of cases	Apoptosis of placental trophoblast cells (number/field of view)	*t*	*P* value
PROM group	108	58.46 ± 11.26	26.152	0.000
Control group	108	21.06 ± 9.70		

PROM = premature rupture of membranes.

### 3.2. Comparison of factors associated with trophoblast cell apoptosis in the placenta between the PROM group and the control group

The relative expression levels of Caspase-1, Caspase-3, NLRP3, and IL-1 proteins, which are associated with trophoblast cell apoptosis, were significantly elevated in the PROM group compared to the control group (*P* < .05). Please refer to Table 2 for detailed data.

**Table 2:** Comparison of factors associated with trophoblast cell apoptosis in the placenta between the PROM group and the control group.

Group	Number of cases	Relative protein expression level of Caspase-1	Relative protein expression level of Caspase-3	Relative protein expression level of NLRP3	Relative protein expression level of IL-1β
PROM group	108	1.32 ± 0.26	1.19 ± 0.30	1.29 ± 0.28	1.23 ± 0.24
Control group	108	0.44 ± 0.17	0.50 ± 0.16	0.52 ± 0.17	0.61 ± 0.21
t		29.440	21.090	24.429	20.204
* P*		.000	.000	.000	.000

IL-1β = interleukin-1β, NLRP3 = nucleotide-binding oligomerization domain-like receptor family pyrin domain containing 3, PROM = premature rupture of membranes.

### 3.3. Comparison of factors associated with trophoblast cell apoptosis in the placenta between full-term and preterm patients in the PROM group

In the PROM group, the relative expression levels of Caspase-1, Caspase-3, NLRP3, and IL-1 proteins, which are associated with trophoblast cell apoptosis, did not show significant differences between full-term and preterm patients (*P* > .05). Please refer to Table 3 for detailed data.

**Table 3:** Comparison of factors associated with trophoblast cell apoptosis in the placenta between full-term and preterm patients in the PROM group.

Group	Number of cases	Relative protein expression level of Caspase-1	Relative protein expression level of Caspase-3	Relative protein expression level of NLRP3	Relative protein expression level of IL-1β
Full term	48	1.31 ± 0.23	1.18 ± 0.28	1.27 ± 0.26	1.22 ± 0.23
Preterm	60	1.33 ± 0.29	1.20 ± 0.26	1.31 ± 0.28	1.24 ± 0.21
t		−0.390	−0.384	−0.761	−0.471
* P*		.698	.702	.448	.638

IL-1β = interleukin-1β, NLRP3 = nucleotide-binding oligomerization domain-like receptor family pyrin domain containing 3, PROM = premature rupture of membranes.

### 3.4. Comparison of pyroptosis-related factors of placental trophoblast cells in patients with different reproductive tract infections in the PROM group

In the PROM group, the relative expression levels of Caspase-1, Caspase-3, NLRP3, and IL-1 proteins, which are associated with trophoblast cell apoptosis, were significantly higher in patients with multiple genital tract infections compared to those with single infections or without infections (*P* < .05). Similarly, patients with single genital tract infections in the PROM group exhibited higher relative expression levels of these proteins compared to those without infections (*P* < .05). Please refer to Table 4 for detailed data.

**Table 4:** Comparison of pyroptosis-related factors of placental trophoblast cells in patients with different reproductive tract infections in the PROM group.

Group	Number of cases	Relative protein expression level of Caspase-1	Relative protein expression level of Caspase-3	Relative protein expression level of NLRP3	Relative protein expression level of IL-1β
Single infection	59	1.22 ± 0.20	1.19 ± 0.26	1.26 ± 0.23	1.21 ± 0.20
Multiple infections	27	1.41 ± 0.19a	1.35 ± 0.23a	1.51 ± 0.21a	1.40 ± 0.18a
Not infected	20	1.00 ± 0.18ab	0.96 ± 0.24ab	1.06 ± 0.22ab	0.99 ± 0.19ab
*F*		26.653	14.550	25.279	26.880
*P*		.000	.000	.000	.000

Note: a, compared with single infection *P* < .05; b, compared with multiple infections *P* < .05.

IL-1β = interleukin-1β, NLRP3 = nucleotide-binding oligomerization domain-like receptor family pyrin domain containing 3, PROM = premature rupture of membranes.

## 4. Discussion

This study showed that relative protein expression levels of Caspase-1, Caspase-3, NLRP3, and IL-1 were increased in PROM group, which correlated with inflammation. Inhibition of NLRP3 may provide a therapeutic target for clinical PROM treatment. The increase of NLRP3 proves that NLRP3 can also be used as a clinical predictor of PROM. Genital tract infections have the potential to disrupt the delicate microbial balance within the reproductive tract. In pregnant women, this disruption can result in adverse pregnancy outcomes, including the occurrence of preterm PROM.^[14,15]^ As a result, timely intervention becomes crucial for this specific group of pregnant women to mitigate the incidence of preterm PROM. Cell apoptosis is a programmed cell death process characterized by the rupture of cell membranes and the subsequent release of inflammatory contents.^[6,16]^ The primary objective of this study is to delve into the mechanisms underlying trophoblast cell apoptosis within the context of genital tract infections, particularly in relation to preterm PROM.

The results of this study demonstrated that the number of trophoblast cell apoptosis in the placenta was significantly higher in the PROM group compared to the control group (*P* < .05). Cell apoptosis is induced under pathological conditions. During apoptosis, small pores appear on the cell membrane, disrupting the concentration gradient and increasing the osmotic pressure. This leads to cellular swelling and rupture, resulting in the release of cellular contents into the extracellular space.^[17,18]^ In addition, cell apoptosis is accompanied by nuclear condensation and degradation of DNA into fragments. In the context of genital tract infections associated with preterm PROM, impaired invasion of trophoblast cells can affect spiral artery remodeling, thereby affecting blood supply at the maternal-fetal interface and leading to placental hypoxia.^[19–21]^ Under the aforementioned conditions, the placenta may secrete certain cytokines that can cause injury to maternal endothelial cells. In such cases, the placental function may be insufficient to meet the nutritional demands of pregnancy.^[22]^ Additionally, genital tract infections that lead to preterm PROM can disrupt the body’s innate immune system. When the body recognizes pathogen-associated molecular patterns during the defense against foreign invasions, it can induce programmed cell death in infected cells, resulting in trophoblast cell apoptosis.

The outcomes of this study have shed light on significant findings. Specifically, the relative expression levels of Caspase-1, Caspase-3, NLRP3, and IL-1 proteins, which are closely associated with trophoblast cell apoptosis, were markedly elevated in the PROM group in comparison to the control group. These findings suggest the presence of cell apoptosis within the fetal membrane tissues of the observed group. Preterm PROM is characterized by structural abnormalities and inflammation in fetal membranes. This condition not only compromises the integrity of the membranes but also exerts an impact on the body’s immune system.^[23]^ In the context of genital tract infection, inflammation can trigger the production of various protein molecules that actively participate in the immune mechanisms of placental membrane tissues.^[24]^ According to a prior study,^[25]^ IL-1 has been identified as an inducer of matrix metalloproteinases (MMPs), and MMPs play a pivotal role in the metabolism of extracellular matrix proteins within fetal membrane tissues. When MMPs are overexpressed, they have the capacity to degrade collagen within the extracellular matrix, ultimately resulting in membrane rupture. Furthermore, in the presence of pathogenic infection or the stimulation of danger signals, the occurrence of preterm PROM relies on fetal membrane cells initiating classical and/or nonclassical pathways of cell apoptosis.^[26,27]^ In these pathways, the activation of Caspase-1 and Caspase-3 promotes the maturation of IL-1 and acts upon the substrate Gasdermin family D protein (GSDMD). The role of GSDMD in microbial-induced inflammatory responses has been extensively explored.^[28]^ Based on the discoveries from study, the robust generation of Caspase-1 and Caspase-3 contributes to the formation of small pores within fetal membrane cells, leading to cellular swelling and dissolution, ultimately culminating in membrane rupture.

NLRP3 is an inflammasome that plays a role in nonspecific immune responses within the placenta. It can be found in various cell types, including fetal membrane epithelial cells, decidual endothelial cells, and trophoblast cells.^[29]^ When fetal membrane cells are exposed to external stimuli, intracellular NLRP3 can form complexes with the adaptor protein ASC and caspase-1 precursor, completing the assembly of the inflammasome and subsequently activating caspase-1. Analyzing the assembly and activation of NLRP3 inflammasomes is a critical step in the pathway of cell apoptosis.^[30,31]^ Based on the results of this study, it is apparent that the levels of NLRP3 increase in fetal membrane tissues following genital tract infection. The primary role of NLRP3 inflammasomes in the pathogenesis of preterm PROM appears to be the promotion of inflammatory factor release, which subsequently leads to cell apoptosis and ensuing inflammatory responses in the placenta and possibly throughout the entire body. Further comprehensive research is needed to explore the precise role of NLRP3 in the pathogenesis of preterm PROM.

Under the influence of pathogenic infections or danger signals, the innate and adaptive immune responses of the body are activated to recognize pathogen-associated molecular patterns.^[32]^ As previously mentioned, the relative expression levels of Caspase-1, Caspase-3, NLRP3, and IL-1 proteins have been closely associated with preterm PROM. The findings from this study also indicate that there is no statistically significant difference in the relative expression levels of these proteins related to trophoblast cell apoptosis between preterm and full-term patients within the PROM group. This suggests that cell apoptosis is present in both preterm and full-term cases of preterm PROM. However, previous studies have indicated that as gestational age progresses, a higher level of cellular apoptosis is required for membrane rupture to occur.^[33]^ The results of this study differ from prior research, emphasizing the need for further investigation.

In recent years, there has been a growing emphasis on conducting in-depth research into cellular apoptosis within clinical practice. Exploring the intricate cascade of events in cellular apoptosis and identifying pivotal targets within this cascade holds the potential for the development of novel approaches to prevent and treat preterm PROM. The findings of this study illustrate that the relative expression levels of trophoblast cell apoptosis-related factors, including Caspase-1, Caspase-3, NLRP3, and IL-1 proteins, exhibit a progressive increase in patients without infection, those with single genital tract infections, and those with multiple genital tract infections. This observation suggests that as the severity of genital tract infection escalates, it promotes trophoblast cell apoptosis, underscoring the critical need for clinical attention. According to a previous study,^[34]^ following genital tract infection, both the immune system and the body’s resistance become compromised. At the maternal-fetal interface, when pathogens invade the reproductive tract, pattern recognition receptors initiate the placental immune response by recognizing components derived from the pathogens. The extent of the signal cascade triggered by pattern recognition receptors can vary based on the degree of infection and the type of pathogens involved. Generally, more severe infections lead to stronger immune responses, heightened cellular apoptosis, and more pronounced inflammatory reactions. Therefore, the expression of trophoblast cell apoptosis-related factors may vary depending on the severity of the infection.

The findings of this study offer valuable experimental evidence that can guide the development of strategies targeting inflammatory factors such as NLRP3, caspase-1, caspase-3, and IL-1 for the prevention and treatment of preterm PROM. Nonetheless, it is essential to acknowledge the limitations of this study. The results may have a certain degree of deviation due to significant individual variations and the relatively small sample size. To enhance the robustness of the findings, future research should consider increasing the sample size, replicating the experiments, and minimizing the influence of confounding factors on the results.

In summary, the expression of trophoblast cell apoptosis and pyroptosis-related factors, including Caspase-1, Caspase-3, NLRP3, and IL-1β, is significantly upregulated in cases of preterm PROM. These findings are associated with the presence of genital tract infections.

## Author contributions

**Conceptualization:** Yunying Qian, Guiying Qian, Haiyan Ni, Danying Zhu, Weiqun Gu, Ximei Cai.

**Data curation:** Yunying Qian, Guiying Qian.

**Formal analysis:** Yunying Qian, Guiying Qian.

**Investigation:** Yunying Qian, Haiyan Ni.

**Methodology:** Yunying Qian, Haiyan Ni, Weiqun Gu.

**Software:** Danying Zhu.

**Supervision:** Yunying Qian, Guiying Qian, Haiyan Ni, Danying Zhu, Ximei Cai.

**Validation:** Danying Zhu.

**Visualization:** Haiyan Ni.

**Writing – original draft:** Yunying Qian, Guiying Qian.

**Writing – review & editing:** Yunying Qian, Guiying Qian, Weiqun Gu, Ximei Cai.
